# Tobacco use and cessation in the context of ART adherence: Insights from a qualitative study in HIV clinics in Uganda

**DOI:** 10.1016/j.socscimed.2021.113759

**Published:** 2021-03

**Authors:** Frances Thirlway, Kellen Namusisi Nyamurungi, Joseph K.B. Matovu, Andrew Kibuuka Miti, Noreen Dadirai Mdege

**Affiliations:** a Department of Sociology, University of York, York, YO10 5DD, UK; b Department of Health Policy Planning and Management, School of Public Health, Makerere University, Kampala P.O Box 7072, Kampala, Uganda; c Department of Community & Public Health Faculty of Health Sciences, Busitema University, Mbale, Uganda; d Department of Disease Control and Environmental Health, School of Public Health, Makerere University, Uganda; e Department of Health Sciences, Faculty of Sciences, University of York, York, YO10 5DD, UK

**Keywords:** HIV, Tobacco, Sub-Saharan Africa, Smoking cessation, Tuberculosis, Biographical disruption, Antiretroviral therapy adherence, Masculinity

## Abstract

Sub-Saharan Africa carries a disproportionate burden of human immunodeficiency virus (HIV). Tobacco use amongst people living with HIV is higher than in the general population even though it increases the risk of life-threatening opportunistic infections including tuberculosis (TB). Research on tobacco use and cessation amongst people living with HIV in Africa is sparse and it is not clear what interventions might achieve lasting cessation. We carried out qualitative interviews in Uganda in 2019 with 12 current and 13 former tobacco users (19 men and 6 women) receiving antiretroviral therapy (ART) in four contrasting locations. We also interviewed 13 HIV clinic staff. We found that tobacco use and cessation were tied into the wider moral framework of ART adherence, but that the therapeutic citizenship fashioned by ART regimes was experienced more as social control than empowerment. Patients were advised to stop using tobacco; those who did not concealed this from health workers, who associated both tobacco and alcohol use with ART adherence failure. Most of those who quit tobacco did so following the biographical disruption of serious TB rather than HIV diagnosis or ART treatment, but social support from family and friends was key to sustained cessation. We put forward a model of barriers and facilitators to smoking cessation and ART adherence based on engagement with either ‘reputation’ or ‘respectability’. Reputation involved pressure to enjoy tobacco with friends whereas family-oriented respectability demanded cessation, but those excluded by isolation or precarity escaped anxiety and depression by smoking and drinking with their peers.

## Highlights

• People living with HIV in Uganda hid their tobacco use from health workers.
• The experience of tuberculosis was the most common trigger to quit smoking.
• Engagement with ‘respectable’ masculinity promoted smoking cessation.
• Engagement with ‘reputational’ masculinity made it hard to quit.
• This model has wider applicability for antiretroviral therapy adherence.

## 1 Introduction

Sub-Saharan Africa carries a disproportionate burden of human immunodeficiency virus (HIV), accounting for more than 70% of the global burden of infection (Kharsany and Karim, 2016). Tobacco use by people living with HIV is higher than in the general population (Mdege et al., 2017; Murphy et al., 2018), including in Uganda, which is the location for this study (Kruse et al., 2014; GATS, 2013). This is a particular concern because tobacco use makes people living with HIV more vulnerable to opportunistic infections (Siddiqi and Mdege, 2016; van Zyl Smit et al., 2010). In Sub-Saharan Africa, the most significant of these is tuberculosis (TB), which has seen a strong resurgence in a population weakened by HIV (Glaziou et al., 2018) and is now the leading killer of people living with HIV (WHO, 2019).

The reasons for continuing higher tobacco use amongst people living with HIV than in the general population are not well-understood but possible factors include socioeconomic disadvantage, other substance use, depression and anxiety, lack of social support, traumatic life events and addiction (Krishnan et al., 2017). Socioeconomic factors are of increasing importance in the lives of people living with HIV in Sub-Saharan Africa; concerns about immediate death have been overtaken by financial problems arising from chronic illness i.e. financial precarity, caring for children etc. (Whyte, 2015). Tobacco use in both Uganda and Sub-Saharan Africa is associated with poverty, low levels of education and agricultural, manual or unskilled work (Sreeramareddy et al., 2014; Kabwama et al., 2016; Murphy et al., 2013). Research on smoking cessation and HIV remains sparse (Diaz and Ferketich, 2018) and there is little evidence of effective interventions for lasting cessation (Pool et al., 2016). Whilst the ‘biographical disruption’ (Bury, 1982; Carricaburu and Pierret, 1995) of receiving a HIV diagnosis and starting ART can itself be a trigger to quit (Edwards et al., 2019, Mitton et al., 2018), the factors which might support a lasting departure from tobacco use are not well understood.

Smoking cessation in the context of HIV is effectively a sub-category of adherence to antiretroviral treatment (ART); in return for their care, people living with HIV are required to ‘work at’ staying well by taking their medication as prescribed and adopting ‘responsible lifestyles’ including the avoidance of alcohol and tobacco (de Kok et al., 2018). ART programmes create a form of ‘therapeutic citizenship’ (Nguyen, 2005), fashioning people living with HIV into empowered, knowledgeable citizens and activists able to self-manage their condition (Russell et al., 2016b; Russell and Seeley, 2010). However, alternative readings have suggested greater patient ambivalence towards ART adherence (Mfecane, 2011) and a therapeutic citizenship tending more towards social control than empowerment (Mattes, 2011; Mattes, 2012; Dilger, 2012; Biehl, 2009 p. 284, Mfecane, 2011).

Gender is a key variable in both tobacco use and HIV: men in Uganda are twice as likely as women to use tobacco (GATS, 2013); women have a much higher HIV prevalence, but men are less likely to get tested and to access HIV care (Gilbert and Selikow, 2011), so HIV clinics are typically 70% female (Mutabazi-Mwesigire et al., 2014). Barriers for the ‘missing men’ of ART include work mobility but also gender norms of men providing food for their families rather than care-seeking (Camlin et al., 2016; Tsai and Siedner, 2015; UNAIDS, 2017) and struggling to reconcile ART compliance and surveillance with ideals of masculine strength and autonomy (Russell, 2019; Matovu et al., 2014). Hegemonic masculinity is often seen as problematic in HIV prevention, for instance as it affects gender power differentials and HIV transmission (Jewkes and Morrell, 2010; Mfecane, 2011). An alternative view proposed by Siu is that contrasting registers of masculinity as ‘respectability’ (including being a good husband and father) or ‘reputation’ (including sexual prowess, alcohol consumption etc.) can either support or undermine ART adherence (Siu et al., 2013; Siu, 2013; Russell, 2019).

The aim of this study was to explore patterns of tobacco use and cessation and facilitators and barriers to cessation among adults receiving treatment for HIV in Uganda, with a view to developing effective smoking cessation interventions. It was carried out alongside a survey of HIV clinic patients and tobacco use (Mdege et al., 2020).

## 2 Methods

Semi-structured interviews were held in 2019 with 25 people living with HIV and attending four HIV clinics in contrasting areas. These were the tobacco-growing areas of West Nile (Northern Region) and Hoima (Western Region), which are known to have high rates of tobacco use (Nzabona et al., 2019), Karamoja, one of the world's poorest areas (United Nations Population Fund, 2018), where snuff is commonly used (Northern Region), and a deprived neighbourhood in the capital city Kampala in the Central Region. We also interviewed thirteen health workers in 13 clinics across Uganda (see Table 1). ART patients who took part were a convenience sample of smokers and quitters enrolled in care and recruited with the help of clinic staff. Patients included 19 men and six women who had been on ART for an average of 6–7 years. Their ages ranged from 28 to 56 with a mean of 42. Twelve were current tobacco users (10 men and 2 women) and thirteen were former users who had quit since their HIV diagnosis (9 men and 3 women). We had difficulty recruiting women to the study and only interviewed six women in total: three snuff users from Karamoja and three sex workers from other regions. All the participants smoked manufactured cigarettes or rolled their own using local tobacco, except for the six participants from Karamoja, who used snuff, and the three sex workers, who smoked a pipe. Ethnic groups represented were Lugbara, Nyoro, Baganda and Karimojong. Socio-economic status was not measured but participants described occupations which were manual, low-paid and often precarious. To protect anonymity, participant locations have been omitted and names changed, or omitted where location is mentioned. Health workers have been identified by region only, and quotes from the sole Eastern Region worker have been grouped with Central Region quotes. Ethics and other regulatory approvals for the study protocol, material and procedures were obtained from the Makerere University School of Public Health Higher Degrees, Research and Ethics Committee (protocol number HDREC 704, approval date June 28, 2019), Uganda National Council for Science and Technology (approval number SS 5073) and the Health Sciences Research Governance Committee at the University of York (approval date May 16, 2019). The research team also obtained permission from the Ministry of Health to access the health facilities.

**Table 1:** Fieldwork locations.

Region	District	ART patients	HIV clinic staff
Central	Entebbe	0	3
	Kampala	7	2
Eastern	Jinja	0	1
Northern	Arua	6	1
	Gulu	0	1
	Lira	0	1
	Moroto	6	1
Western	Hoima	6	1
	Kabarole	0	1
	Mbarara	0	1
		**25**	**13**

Following a training day to introduce the study, research assistants experienced in qualitative research carried out, transcribed and translated the interviews between October and December 2019. Interviews with people living with HIV were conducted in the relevant local language (Lugbara, Orunyoro, Luganda or Ng'akarimojong), whilst interviews with clinic staff were in English. Interviews with patients lasted around 45 min each and were held in private rooms at the clinics or in community locations. We drew on the literature on tobacco use and cessation to prepare a topic guide with questions on household composition, employment, family tobacco use and cessation history, health status, alcohol and drug use, years on ART and hopes and expectations for the future. The purpose of the study was explained to the participants, who signed a consent form to confirm their agreement to take part. They were provided with refreshments, as well as transport costs if they made a special trip. Interviews were audio recorded and supplemented with additional data from interviewers' notes made at the time, a group debriefing and individual debriefings with the research assistants who had conducted the interviews. Social science analysis requires interpretative depth (Panter-Brick and Eggerman, 2018) rather than a paraphrase of what people say (Fassin, 2013 p. 122); the first two authors conducted thematic and narrative analysis informed by regular meetings of the authorial team; themes were developed and added to through regular reference back to the transcripts and theoretical literature. We summarised data in two spreadsheets, one for patients and one for health staff. This format enabled us to compare experiences by reading down each thematic column, whilst preserving each narrative as a unit by reading along each row (Riessman, 2008 p. 12, Ezzy, 2000).

## 3 Findings

### 3.1 *‘They come saying they've stopped, or they just pretend when they come here’:* hiding tobacco use from health workers

Identifying clinic patients with current or past tobacco use proved a challenge. Our experienced interviewers reported that ours was one of the hardest studies they had recruited to; patients were reluctant to admit they used tobacco because they did not want clinic health workers to find out and *‘bark [shout] at them’* (interviewer feedback). Health workers confirmed that patients concealed their tobacco use once they realised it was frowned upon: *‘They don't have information that it's not allowed, but if we give them information, you realise that the next visit, he comes back when he has not smoked. He also fears the doctor to know that he has smoked … but when you meet him outside the facility, he could be smoking or even drunk’,* a Central Region health worker told us. Another said: *‘They come saying ‘I've stopped’ or they just pretend when they come here, because they know that we are going to ask about it. When they are coming they try to chew something so that (we) don't smell it, and to avoid being asked questions’.* We did not ask about this in the patient interviews so we have no direct quotes from tobacco users, but we can usefully draw on Janet (36)'s account of concealing her drinking: *‘The health workers here, when we come for refills, they tell us that it is not good to smoke, and even take alcohol – but me, I lie to them about alcohol … I think some people still smoke, you can't know: like how I take alcohol but I lie [laughs]’.* Janet had overcome serious illness and poverty with the help of her family, and had a catering job which she supplemented with sex work with a few regulars from her time as a street sex worker. Her interview transcript is full of laughter, and our interviewer described her as *‘so lively and ready to share whatever I asked’*. Janet told us: *‘Smoking is fun, especially at night when you are many, you are having fun in clubs as you take some beers … I chose my health over that fun, but I can still have fun with the alcohol, so I don't mind’.* Later she reminded the interviewer: *‘You better not share anything we have discussed here, because I don't want any health worker to know about this, because they think I don't take alcohol’*. Women participants were particularly hard to find, and both health workers and participants themselves confirmed that women concealed their tobacco use: ‘*Women don't open up to tell you that they are using tobacco. Mostly the people we get are men because they are the ones who open up easily’,* one Central health worker said. According to a Western Region health worker*: ‘[Smoking] is so common in men, because even according to our culture, when a woman smokes, people comment negatively, but with men, the public think that it's ok’.* Smoker Christine (40) told us that she and other sex workers had ‘*a hidden place around town where we all gather and smoke’.* She also smoked at home, but told us: ‘*I endeavour to lock the door and smoke from inside the house; I would not want my neighbours to know my habit [laughs]. This place is not like Kampala where you can smoke the pipe openly without anyone's interference’*.

The context of non-disclosure was that patients were told tobacco use could make their medication less effective and expose them to a higher risk of opportunistic infections, both when they started on ART and again at regular clinic health education sessions. During the course of the interviews, participants living with HIV agreed that tobacco was harmful, except for one patient and one health worker in Karamoja who were unsure about snuff. Whilst acknowledging the dangers of tobacco in general terms, two smokers in the Northern Region argued that they had suffered no ill effects and told us about elderly people who still smoked, the ‘defiant ancestor’ or ‘Uncle Norman’ argument (Davison et al., 1991; Balshem, 1991). Health workers repeated the message about tobacco harm in the hope that patients would: ‘*internalise it and after realising it has become a song from me, they decide to stop’*, (Western Region health worker). However, there was no support to quit: *‘We are not told how we can stop smoking or what we can do to stop’*, Mukasa (46) said. It was also clear that health workers associated both alcohol and tobacco use not just with opportunistic infections, but also with poor ART adherence, and specifically with forgetting to take ART medication. One Northern health worker told us tobacco use *‘may predispose you for poor adherence, you may be a poor adherent. You will love tobacco, you cannot do without it, therefore you will opt out from taking your drugs regularly’.* Another said: *‘If they're taking alcohol you can forget with time your medications, and if you're smoking, it also has effect on you.’* Health workers did not explain exactly how tobacco led to forgetfulness, but described smoking and drinking as being part of a lifestyle which was incompatible with taking antiretroviral drugs (ARVs). A Central health worker described the ideal attitude as general abstinence; patients should be saying: *‘For me, ever since I started taking ARVs, I stopped boozing, I stopped loving men, or women, I stopped sniffing [taking snuff] or smoking cigarettes, so my life is my ARVs’*.

Tobacco users in the Northern Region were more open about their use. In Karamoja, taking snuff was the norm. As a health worker told us: *‘Almost everyone smokes or takes snuff … so almost half of the village is snuffing while having HIV, and half of the village is not living with HIV but is sniffing’* [sic]. Local beer seller Santa (35) said: ‘*I smoke [inhale snuff] anywhere, even when in a meeting and I get the urge to, I get it and smoke. I have nothing to hide’.* In the Lango, Acholi and West Nile areas, cigarette smoking was common and was usually disclosed, at least by men. As one health worker said: *‘Patients normally tell you if they are tobacco consumers or not … people who have HIV are always consumers of alcohol and smoking’*. Asked if patients *‘freely put up their hand’* to say they smoke, another health worker told us: *‘They are free, they can put their hands up because they feel smoking is not a bad thing, so for them they feel it's not bad to smoke’.* We had no difficulty finding tobacco users in the West Nile area, but it was harder to locate former users; patients who were on file as having given up tobacco often turned out to be smoking again. Participants' status as smokers or quitters was never definitive. With one exception (snuff user Santa had never tried to quit), the tobacco users we interviewed had all been quitters at some point. Some had stopped using tobacco for as long as six months or a year before relapsing. As one health worker in the Western Region pointed out: *‘Some clients tend to resume their risk behaviours, especially after completing their treatment for TB’*.

### 3.2 *‘If you are not smoking with your friends, they may say haha, you want to isolate yourself’*: using tobacco with friends and colleagues

The barriers to quitting tobacco which we explore in the following sections were mentioned by roughly equal numbers of smokers and quitters. These barriers were sociality (14 mentions), stress or worry (11 mentions), the use of tobacco in cold weather (9 mentions) and the availability of free tobacco (8 mentions). The exception was addiction or habit (15 mentions), which was referred to by all current users as part of their justification for smoking but only by three former users. Tobacco users spoke of being addicted and of cravings or the urge to smoke. They felt something was missing, or were *‘like one in need of some water to drink’*, or felt their nose *‘itching and wanting snuff’*. Using tobacco with family, friends or co-workers (sociality) was the next most common reason given by participants for being unable to quit or for relapsing. For farmer Ronald (53): *‘When I am with friends and my relatives and see them smoking, I also get the urge of smoking tobacco’.* A Northern health worker explained: *‘Tobacco smoking is a kind of social linkage for most people, you get your friends, you share together. Even if somebody has a small stock, you give me, they are not selfish. It's a kind of social way of saying I love you and the other things. So if you are not smoking with your friends, they may say haha, you want to isolate yourself.’* Alcohol and tobacco were both linked with sociality: *‘For men, they feel it's part of their leisure to smoke and drink,’* a Northern health worker said. ‘*Leaving drinking alcohol is the only change that would help me quit, because once I take alcohol, snuff should also be near’,* casual labourer Okot (56) told us. Farmer Florence (39) agreed: *‘When I would go to the gardens to work, the time they bring [local beer] and that is the time people start smoking, [local beer] goes hand in hand with smoking snuff’.* Participants mentioned drinking bottled beer, locally-produced beer and waragi (Ugandan gin). Like many women in Karamoja, Santa (35) made a living selling the local beer (Dancause et al., 2010), which she also drank whilst taking snuff.

Tobacco use was normative in some occupations: *‘The kind of work I do is construction, so most of the people I work with do smoke; this is what has made it hard for me to successfully quit’* (Moses, 59), and: *‘I learnt smoking from the sand mine, I was fourteen years old’* (Kizito, 47). A Western Region health worker told us: *‘It's so common among some occupational groups like bar workers, people who work at night including boda [motorcycle taxi] riders, because of night coldness'.* Seven participants with backgrounds in the military, the construction and fishing industries, farming, security and sex work spontaneously mentioned smoking to keep warm at work. Samuel (50) started work as a fisherman at fifteen: *‘They used to say cigarettes help in removing coldness: I continued that in a bid to fight the cold, till it became a habit.’* Christine (40) told us: ‘*There is no female sexual worker who does not use tobacco, because it attracts men towards us and that is how we earn a living’*. Janet (36) used cannabis and shisha with her customers, and: *‘In order to get more clients, we would also smoke the pipe where we would put herbs – kayaayaana* [or kayayana: used by traditional healers as a love potion] *– some tobacco leaves and marijuana, this would make us drunk too so that we don't get shy when we go to work’.*

Participants linked tobacco with physical strength. Farmer and former tobacco grower Benon (36) said: *‘We were always told that cigarette smoking makes one more intelligent and energetic [by] those who used to smoke the tobacco, including my parents'.* Subsistence farmer Ronald (53) added: ‘*When I work and get tired, I smoke and feel relieved and even work more*.’ Brick-maker Kizza (42) sang us the British American Tobacco (BAT) Sportsman brand radio jingle from the 1990s: *‘Sportsman Ye, Ssebo’* [what a man!], also a prominent slogan in Kampala at that time (Baingana, 2005 p. 106). Five out of six participants from the Western Region had worked in the tobacco industry or grown tobacco, and the West Nile research area was home to a high-profile Pan-African Tobacco Group (PTG) processing plant (Tobacco Reporter, 2017).

Using tobacco with family was also a barrier to quitting; builder Hamuza (38) told us: *‘I was born seeing my dad smoking’* and Mukasa (46) complained that: *‘Family and friends prefer being with me like this as a smoker’.* All but four of the twenty-three people for whom we had these data had grown up with at least one parent who used tobacco. Mujib (50) told us his grandmother would*: ‘send me to the shop to buy for her some sticks of cigarettes very often, that was how I learned to smoke, I was eleven years old’.* Mukasa (46) was sent for cigarettes by his brothers and since they had no matches, they told him to *‘light it from the shop and then puff by inhaling so that the cigarette continues to light until I reach home and give it to them’.* For shopkeeper Alice (34): ‘*The tobacco leaves that my grandmother used to have because she would cultivate were the ones I would grind to powdered form to make snuff, and gradually I caught up on smoking; that was at the age of about eight years’*.

The common practice of sharing and gifting tobacco made it easily accessible; eight participants referred to free tobacco making it hard to quit and easy to relapse. Construction worker Masaba (40) started smoking in the army: *‘We used to share cigarettes as a group, so most of what I took was free. Some of my colleagues would collect from the gardens and bring and share with us’,* he said. Asked how much he spent, casual labourer Evarist (38) said: *‘Not so much, like you know group smoking, you may not spend any money but your colleagues give you’*. If he tried to quit, his co-workers *‘simply give you that – have this’.* Beer seller Santa (35) said that: *‘A number of times when I run broke are the only times that make me want to quit, but then again since snuff is held by many and is easily shared, it becomes easy for me to get back to smoking [using snuff]’.*

Self-assessed cigarette consumption was generally less than five sticks a day, and most smokers bought single cigarettes. Builder Mukasa (46) smoked three a day: *‘I use 600 shillings daily on cigarettes, each stick of cigarettes is for 200 shillings in the retail shops around the area where I stay’*, he told us. Mechanic Umaru (44) reduced his smoking after his HIV diagnosis and his daily spend was: ‘*Only 200 shillings. Because I take one stick which costs only that amount, from some retail small shops’.* Thirteen of the eighteen current or former smokers of manufactured cigarettes in our study favoured the Sportsman brand which BAT targets at low-income smokers (Mbabazi, 2019). Kizza (42) remembered: *‘vehicles with PA systems telling people about cigarettes and giving out free samples’,* and Mukasa (46) said: *‘promoters were distributing these cigarettes to people for free’*. Mujib (50) also recalled leaf merchant Alliance One making: *‘promotions of tobacco products where they give people who win different gifts like free T-shirts, phones and so forth … they even give people free packets of cigarettes.*’

The three sex workers we spoke to all smoked pipes, we were told that many middle-aged women secretly smoked pipes *(emindi*) of herbs obtained from traditional healers alongside tobacco to achieve desired outcomes or *‘blessings’*. In the Karamoja Region, participants bought small polythene bags of local snuff, known as ‘*kuuli*’ and spent between 300 Ugandan shillings a day and 300 a week. Participants in the West Nile area either smoked manufactured cigarettes or used locally produced tobacco to roll their own: *‘That one I cannot spend some money on it, I just get it free from friends’*, one man told us. Another added: *‘I smoke the locally rolled tobacco with a paper …. I do smoke six of them in a day and buy at 200 shillings … if I am alone, I smoke it for a week but when (I) am with my brothers, we smoke it for two to three days … cigarettes are everywhere and it's affordable, even if I don't have money, my friends can give me for free … the manufactured one is very expensive and we cannot afford that one’*. One of the Kampala participants also smoked self-rolled tobacco *‘if I do not have money to buy the manufactured type’*.

### 3.3 *‘It was this sickness of TB that made me to leave smoking, since it made my chest pain so much’*: giving up tobacco after tuberculosis

The experience of acute illness and especially TB was mentioned most frequently as a trigger to quit (16 mentions), followed by social support to quit (10 mentions), which we explore in section 3.4. Janet (36) told us how, after she left her abusive husband and turned to sex work to survive, she fell ill and was diagnosed with HIV and TB: *‘I became so sick to the extent of being admitted to hospital … I used to hear that [smoking] causes harm but I thought it was not so severe until I suffered from TB - now I know’.* Both the disease and the treatment were unpleasant, as clothes trader Arthur (30) told us; *‘It was this sickness of TB that made me to leave smoking, since it made my chest to pain so much … I feel a bit better these days except when I take the TB drugs I feel some pain, which makes me very uncomfortable’.*

Some participants attributed their decision to quit to the combined experience of HIV and TB. Sex worker Stellah (41) said: ‘*I realised that I could not sustain myself on two different treatments of ARVs and TB, so I knew it was easier to avoid catching TB again, to prevent myself from such a burden.*’ Unemployed security guard and farm worker Samuel (50), hospitalised for nine months, explained: *‘The acquisition of this sickness (HIV) is the one that made me quit smoking … I sat and imagined how I was going to continue smoking with all these complications, and yet this TB is caused by this smoking. That is why I totally decided to quit.’* Florence (39), a single mother engaged in farming and casual work to provide for her five children, stopped using snuff after she contracted HIV and TB: ‘*I realised that when I would take tobacco I would feel sick … so I said to myself ‘mmm, I think I'm spoiling my body, my health’ – I then decide to leave smoking [using snuff]’.*

Many of the participants who had experienced TB quit both tobacco and alcohol as part of a general resolve to improve their ART adherence. At HIV diagnosis, all participants except one had been drinkers, but all those who quit tobacco as well as more than half the continuing tobacco users claimed they had given up or reduced their drinking. Masaba (40) told us: *‘If I had not quit alcohol and cigarettes, I wouldn't be alive today’*. Arthur (30) was in TB treatment at the time of our interview and had recently quit tobacco and alcohol for this reason.

### 3.4 *‘My wifesupportedme, she told me you can make it’*: quitting tobacco with family support

Ten participants mentioned the support of family and friends as a key factor in their quitting tobacco. Brick maker and former tobacco-grower Kizza (42) told us his smoking cessation ‘*was through my wife's support … she even reached an extent of chasing away some of my friends who used to smoke, and called them bad company. She would monitor me wherever I would go’.* George (42) said: *‘My wife* *supported* *me, she told me you can make it, and we even save that money for the children’*. Street salesman Arthur (30) told us: *‘Because of my sickness, I cannot go back to smoke, and even people around me [family] will not allow me to go back to smoke’*. Shopkeeper Alice (34) described how she stopped using snuff: ‘*My children talked to me and said ‘mama, you are smoking [using snuff], you are even taking alcohol, do you really want to die and leave us?’ then I thought over this for some time and decided to quit, but it was a gradual process'*.

Family support for smoking cessation was key in a context where families were large (nearly half the participants lived in households of six or more) and hopes for the future centred on providing for them. A Northern region health worker advised smokers*: ‘We tell you the effects (tobacco) may have on you, looking at you being a responsible family head, bread earner, what this will do to you if you cannot stop it’.* Farmer Godfrey (50) told us he wanted: *‘the future of the family to be fine, and also build some permanent houses for them*.’ Builder Hamuza (38) wished that: *‘God can give me more life and continue working so that I construct a permanent house for my family’*. Farmer Ronald (53) hoped *‘to stay healthy to up bring my children until they complete their education’.* Sex worker Stellah (41) hated her job, but told us: *‘It is my income that helps me to feed my [five] children, pay school* *fees* *for them as well as pay rent and all these other needs’.*

Participants who were more isolated appeared more likely to struggle with smoking cessation; only one of the six who lived alone had quit. Evarist (38), who was separated from his wife and child, told us: *‘I really want to quit but it's not easy, this poverty and groups of people that I move with since at home I don't have any company, so I meet with my friends for company.’* The uncles who brought him up had both died, and he survived on irregular casual work. Construction worker Moses (59) told us: *‘I tried and quit for some time but I later failed … most of the people I work with do smoke, this is what has made it hard for me to successfully quit’.* He lived alone and drank spirits every day.

### 3.5 *‘I work so hard yet I earn little’*: using tobacco to escape depression linked to food insecurity

More than half the participants described using tobacco to escape anxiety and depression caused by food insecurity and the struggle to support themselves and their families. All but one participant mentioned money concerns, and this was generally money for basic needs, namely feeding their families and sending their children to school. Interviewers described many participants as *‘full of thoughts’*, and noted that several asked them for money for their families. Construction worker Kizito (47) told us he smoked ‘*to* a*void thoughts - thoughts about many things including money’.* Casual labourer Evarist (38) smoked because of: *‘Okuwummuza ku birowoozo - dealing with stress. I work so hard yet I earn little, I spend whatever I earn, so I decide to smoke; I feel less stressed when I smoke.’* Yakobo (45) was too ill to work: *‘My problem is I have children, I have my family and now am thinking about them. There is not work and I just stay without getting any money and yet they want food’.* Carpenter Mujib (50) cried as he told us his work did not pay enough to feed his family: *‘Whenever I think about my children … I wonder if I will be able to provide for them’.* Farmer and security guard Benon (36) said: *‘Depression is mostly because of financial constraints. A situation may arise when it requires money and you get depressed. Currently my child was sent away from school* [Benon could not pay the fees] *and I got depressed’.* A Central region health worker agreed that: *‘When it is the period for paying school* fees*, there are some cases [of depression] that come as a result of this’.* A Western Region health worker added: ‘*Majorly these people are having issues like depression, stress, desperate, lost their marriage or jobs thus resorting to alcohol or drug abuse*.’ Health workers from the Lango and Acholi areas also mentioned poverty linked to armed conflict and displacement into refugee camps in Northern Uganda. Sixteen participants mentioned family bereavements: George, Mujib and Santa were brought up by relatives following their parents' deaths. Masaba, Kizito, Arthur and Ronald had lost their wives. Masaba, Godfrey, Yakobo, Kizza, Florence and Alice had lost adult siblings. Christine had lost her parents, grandparents and husband at a young age and Alice lost two infant children.

Several participants described tobacco as a food substitute; Innocent (40) told us: *‘When I am hungry and I smoke, my body feels stronger thereafter and the hunger even disappears … if they can help people fight hunger, then they can stop smoking*.’ Builder and former tobacco factory worker Mukasa (46) added*: ‘If I can afford to get two cups of milk a day, I can quit smoking because I believe milk can substitute my thirst for cigarettes’.* Some participants were unable to afford the food they were supposed to take with their twice-daily medication. Casual worker Evarist (38) was told he *‘wasn't taking the drugs well … sometimes I would not have food yet one needs to eat before taking it, you cannot take it without food because it is very strong’*. A Northern health worker told us that because of poverty, *‘You can find someone who even fails to feed himself in a day two-three times, and instead eats once a day … the majority of our unsuppressed clients are children, we don't have food to eat so they tell us how can they take [ARVs] away when there is no food to eat’.*

## 4 Discussion

Our first finding was that people living with HIV hid their tobacco use from health workers. This has important implications both for supporting cessation and for interpreting prevalence data unsupported by biological validation measures (see for instance Mitton et al., 2018). Although studies rarely account for it, under-reporting of tobacco use is not new: up to a third of self-reported former smokers (and particularly women) in Sub-Saharan Africa have been found to have cotinine levels or exhaled monoxide levels indicating that they still used tobacco. This was the case both for the general population (Jagoe et al., 2002) and people living with HIV (Kruse et al., 2014; Elf et al., 2017). Our study is the first to explore the context of non-disclosure: in an environment where patients were advised to quit tobacco but no support was available to do so, there was little incentive to admit use. It was easier to avoid moral censure and perform the role of the ‘perfect adherer’ (Stadler et al., 2016). Since ART initiation on clinic entry only became universal in Uganda in 2016, some patients may also have feared that disclosure of substance use could affect their eligibility for ART (Adong et al., 2019).

How does the concealment of tobacco use by people living with HIV relate to the idea of therapeutic citizenship? Looking at smoking cessation as a proxy for regimes of ART adherence more generally, we found little trace of the patient empowerment which originally formed part of original concept. Russell and colleagues explain how Uganda's limited political space for activism distinguishes it from some other African contexts where people with HIV have agitated for treatment and therefore feel a political responsibility to adhere, but those authors do find an emergent (male) HIV community taking responsibility for their own health. They suggest the greater social control of ART patients observed by Mattes might be explained by their lower social status compared to Russell's own study participants (Russell et al., 2016a; Mattes, 2012). This might explain why our findings were closer to Mattes's; the association between disadvantage and tobacco use means that the people we interviewed were some of the poorest and most precariously employed in Ugandan society, pointing to the need for social scientists to relate differences in ART experience and therapeutic citizenship more clearly to issues of class and power.

Our findings are consistent with studies suggesting that men at least are more ambivalent towards ART adherence than the therapeutic citizenship ideal suggests, and that smoking and drinking are key stumbling blocks. Those men most anxious to follow leisure norms of alcohol and tobacco use are most likely to drop out of ART (Siu et al., 2013 pp. 218–225); ‘*These drugs deprive us of fun’*, says one man quoted by Mfecane as he struggles with expectations that he will abandon smoking and drinking and become a ‘responsible’ patient (Mfecane, 2011). Whilst the idea that masculinity can be bad for your health is not new, Siu's ground breaking analysis suggests that engagement with different masculinities (Connell, 2005) can result in men either embracing or rejecting health strictures (Siu et al., 2013). A ‘reputational’ register of masculinity privileging physical strength and ‘compulsory leisure’ discourages ART engagement, but family and societal expectations to be a family provider and ‘respectable’ role model encourage it (Siu et al., 2013).

Siu's notion of contrasting masculinities illuminates our data and provides us with the basis for a model of barriers and facilitators to smoking cessation: for our participants, sociable, ‘reputational masculinity’ made it difficult to quit (Finding 2). It involved pressure to share and enjoy tobacco with others, occupational norms of tobacco use and a legacy of advertising messages connecting tobacco with physical strength. On the other hand, family support to quit bolstered adherence through an appeal to respectable masculinity; participants described tobacco cessation as being both explicitly supported by family members and motivated by their own desire to provide for their families (Finding 4). However, men who were socially isolated or in precarious employment could not position themselves successfully within the register of respectable masculinity, which involved supporting themselves and ideally a family. Instead, they used tobacco to escape anxiety and depression caused by food insecurity, falling back on reputational masculinity and getting validation through smoking and drinking with their peers (Finding 5).

The fact that tobacco and alcohol use emerged as subcategories of ART adherence means our model is potentially relevant to ART adherence more generally. Barriers to ART adherence in Sub-Sarahan Africa include food insecurity (Hardon et al., 2007) and depression (Mayston et al., 2012; Cole and Tembo, 2011; Uthman et al., 2014); in fact food insecurity itself leads to depression (Singer, 2011; Kidia et al., 2015; Tsai et al., 2012), often expressed in African settings as ‘thinking too much’ or having ‘too many thoughts’ (Okello, 2006; Kidia et al., 2015; den Hertog et al., 2016). Isolation and lack of social support are barriers to ART adherence (Croome et al., 2017; Heestermans et al., 2016) as well as smoking cessation (Chandola et al., 2004; Choi and DiNitto, 2015). Our model of barriers and facilitators to smoking cessation is also potentially applicable to disadvantaged smokers more generally in that it brings contradictory factors (for instance, tobacco use linked to both sociality and isolation) (Hiscock et al., 2012; Barbeau et al., 2004) into a logical sequence rooted in the life course and historically contingent gender roles (Connell, 2005 p. 27).

Turning to women's tobacco use, we found that registers of respectability and reputation were not exclusive to masculinity; women also contrasted the ‘fun’ of smoking with friends, and the decision to quit in order to stay healthy for their children (McDermott et al., 2006; Nichter et al., 2007). We suggest that the respectability register was more strongly socially sanctioned for women than for men, so that women were less likely to use tobacco or at least, admit to use. Men were more invested in the reputational register, and therefore had to make greater behaviour changes than women to comply with ART requirements (Russell, 2019). The reputation/respectability dualism used by Siu originates in the anthropology of the Caribbean (Wilson, 1969), but whilst Wilson associated men with reputation and women with respectability, Miller argued that gender was not the basis of this dualism (Miller, 1994 p. 259). The very small number of women involved in our study does not allow us to theorise further in relation to women and tobacco.

How did study participants give up tobacco in practice? Whilst receiving a HIV diagnosis and starting ART can be a trigger to quit tobacco, equating the onset of chronic illness with significant biographical disruption involves the ‘assumption that illness enters lives previously untouched by crises’, whereas HIV life trajectories are often ‘characterised by a continuous stream of different crises including violence, poverty and death’, so that the HIV diagnosis itself is not as big a disruption as might be thought (Wouters and De Wet, 2016 p. 537). In the era of HIV as a chronic rather than acute illness, we found that it was the experience of TB – albeit linked to HIV - which threatened the lives of people living with HIV and therefore operated as a biographical disruption enabling the transition from current to former tobacco user. Even then, abstaining from tobacco was not definitive, and some participants continued or reverted to tobacco use after recovering from serious illness. Once their health status improves, many men living with HIV revert to their previous lifestyles (Mfecane, 2011).

How can health policy and practice support people living with HIV to give up tobacco as an integral part of ART adherence? Patient-centred care in the HIV clinic has to be balanced against the need to probe about ART adherence (de Kok et al., 2018), but our first finding suggests that more patients would disclose tobacco use if this gave them access to structured cessation support rather than exposing them to ‘scolding’ by health workers (Russell, 2019). It is also important to have ongoing support available for those who quit tobacco when they first start ART or when they contract TB, but who relapse to smoking once their health improves. Our second and fourth findings on sociable smoking and support to quit suggest that peer support groups and testimonies of successful quitters could potentially confirm quitters in their performance of family responsibility and prevent relapse, enabling men in particular to assume a positive gender role (Dilger, 2012 p. 84, Mburu et al., 2014). To address Finding 5, some of the fundamental causes of anxiety and depression i.e. food insecurity and poverty should be addressed through livelihood support initiatives (Tsai et al., 2012), and tobacco users screened and treated for depression. Following our third finding and given the high incidence and life-threatening nature of TB among people living with HIV in Sub-Saharan Africa, HIV staff training and health education materials could usefully focus on the link between tobacco and alcohol use and TB (Jackson-Morris et al., 2015; van Zyl Smit et al., 2010; Harling et al., 2008; Necho et al., 2021). Stricter tobacco control measures would also support people living with HIV in quitting tobacco and decrease the chances of their relapsing to smoking. We saw how the legacy of tobacco advertising and the continuing visibility of the tobacco industry in specific regions contributed to beliefs about tobacco providing strength, combatting the cold or substituting for food; the ready availability of single cigarettes in defiance of the Tobacco Control Act, 2015 made smoking affordable. Tax increases resulting in price increases are one of the most effective ways of decreasing tobacco use prevalence, especially if tax is structured to eliminate wide price variation which allows tobacco companies to target low-income smokers with cheaper products (Laverty et al., 2020); new tax measures could usefully be considered in order to decrease smoking prevalence both amongst people living with HIV and the wider population (Ntale and Kasirye, 2018).

## 5 Limitations

The fact that current and former tobacco users were reluctant to disclose their status meant that in all regions except the North, we relied on health workers to help us recruit those already known to them. Since health workers only probed for tobacco use in cases of poor adherence or suppression, the participants they found for us may have been those with more complex medical histories. However, whilst there has been very limited research on tobacco use and cessation by people living with HIV in Sub-Saharan Africa, our findings are consistent with the substantial literature on ART adherence and on tobacco use more generally, which informed all stages of our analysis. A more significant issue is the lack of women in our study; this and the general lack of research on women's tobacco use in Sub-Saharan Africa has limited our ability to generate theory in relation to tobacco use and cessation among women. Finally, whilst we consider one of the strengths of our study to be its geographical spread, we are conscious that we have not done justice to the very different patterns of tobacco use which prevail in different regions; in particular, snuff use in Karamoja needs more research, as does pipe (emindi) smoking by women in other regions.

## Funding

This study was part-funded by the 10.13039/100010269Wellcome Trust [ref: 204829] through the Centre for Future Health (CFH) at the 10.13039/100009001University of York. NDM is grateful for salary support from the Tobacco Control Capacity Programme (grant MR/P027946/2) supported by 10.13039/100014013UK Research and Innovation (UKRI) with funding from the Global Challenges Research Fund.

## CRediT author statement

NM led and coordinated the wider study of which this forms part. NM, KN, FT and JM participated in the conception and design of this study, KN supervised data collection by a team which included AM. FT and KN analysed the data and FT drafted the initial manuscript. All authors commented on earlier drafts and approved the final manuscript.

## Author contributions

NDM led and coordinated the wider study of which this forms part. NDM, KNN, FT and JKBM participated in the conception and design of this study, KNN supervised data collection by a team which included AKM. FT and KNN analysed the data and FT drafted the initial manuscript. All authors commented on earlier drafts and approved the final manuscript.
